# Mature Rice Biomass Estimation Using UAV-Derived RGB Vegetation Indices and Growth Parameters

**DOI:** 10.3390/s25092798

**Published:** 2025-04-29

**Authors:** Mengguang Liao, Yun Wang, Nan Chu, Shaoning Li, Yifan Zhang, Dongfang Lin

**Affiliations:** 1 Sanya Institute of Hunan University of Science and Technology, Sanya 572024, China; liaomengguang@163.com; 2 College of Geoscience and Spatial Information Engineering, Hunan University of Science and Technology, Xiangtan 411201, China; 22012001011@mail.hnust.edu.cn (Y.W.); lsn@hnust.edu.cn (S.L.); lindongfang223@163.com (D.L.); 3 National-Local Joint Engineering Laboratory of Geo-Spatial Information Technology, Hunan University of Science and Technology, Xiangtan 411201, China; 4 Land Satellite Remote Sensing Application Center, Ministry of Natural Resources, Beijing 100048, China; zhangyifan@lasac.cn

**Keywords:** rice, UAV imagery, above-ground biomass (AGB), RGB vegetation indices, growth parameters, feature fusion

## Abstract

The biomass of rice at maturity serves as a vital indicator for assessing overall productivity, and its accurate estimation holds significant importance for ensuring food security and promoting sustainable agriculture. To improve the precision of current biomass estimation methods for mature rice, this study employed support vector regression to integrate RGB vegetation indices from rice canopy images with growth parameters, thereby developing a biomass estimation model. The model was validated by applying it to the experimental area. The results indicated that screening RGB vegetation indices and combining them with growth parameters enhanced estimation accuracy. Specifically, the model integrating RGB vegetation indices (g, RGBVI) with rice plant height and moisture content demonstrated high estimation accuracy (*R*^2^ = 0.78, *RMSE* = 0.32 kg/m^2^). The absolute difference between the estimated and measured biomass values ranged from 0.15 to 0.39 kg/m^2^. Additionally, the estimated biomass showed a strong correlation with yield (*R*^2^ = 0.86), with a fitted equation of *y* = 0.04*x* + 0.59. These results suggest that the model is reliable for large-area estimation of mature rice biomass. However, the degree of rice maturity and the lodging phenomenon were identified as the primary factors influencing the precision of model application. Overall, integrating RGB vegetation indices of the rice canopy, obtained via UAV-based remote sensing technology, with growth parameters provides an effective method for estimating mature rice biomass and offers a valuable reference for efficient yield estimation.

## 1. Introduction

Rice is one of the most important food crops globally, serving as the staple food for nearly half of the world’s population and playing a crucial role, particularly in Asia [1,2]. China leads the world in both rice production and consumption, accounting for approximately 30% of the global total in both categories [3]. Consequently, rice production is intricately linked to China’s food security, economic development, and social stability. Above-ground biomass (AGB) of rice plants is a key indicator of its growth status and strongly correlates with final yield [4]. At the maturity stage, rice biomass typically includes grain weight, which serves as a critical parameter for evaluating overall crop productivity [5,6,7,8]. Therefore, accurately estimating rice biomass at maturity is essential for ensuring food security, achieving sustainable agricultural development, and supporting data-driven decision-making in national food policy formulation.

Traditional methods of measuring rice biomass typically rely on destructive sampling, which is not only inefficient but also fails to meet the monitoring needs of large areas. Given the current situation of population growth and increasing land resource constraints, efficient new ways of agricultural development are particularly important [9]. In recent years, UAV-based remote sensing technology has been widely applied to estimate crop biomass due to its high flexibility, high spatial-temporal resolution, and ease of operation. The mainstream approach involves analyzing the relationship between spectral indices from UAV remote sensing imagery and rice biomass, using various techniques such as linear regression and machine learning to construct biomass estimation models, which have demonstrated good estimation results [10,11,12,13,14,15,16,17]. However, the physiological and spectral properties of rice exhibit significant stage-dependent heterogeneity. During the early growth stage, the canopy leaves are relatively sparse and have a low overlap rate, making them more sensitive to spectral indices. At this point, there is a notable correlation between biomass and spectral indices. In contrast, during the later growth stages, spectral indices exhibit saturation, leading to a decline in their correlation with biomass [18,19]. This limitation is exacerbated at maturity, where leaf yellowing and structural collapse further reduce spectral sensitivity. For instance, Duan et al. partitioned the rice growth cycle into pre- and post-heading phases, reporting *R*^2^ values of 0.71 and 0.08 for biomass–vegetation index relationships in these phases, respectively [20]. Similarly, Jiang Qi et al. developed a biomass estimation model for rice across its entire growth period based on multiple spectral indices, discovering that the coefficient of determination for models constructed at maturity ranged from 0.01 to 0.25, indicating poor fitting [21]. Zheng Hegang et al. utilized support vector regression methods to establish biomass estimation models for rice at various growth stages based on hyperspectral indices and vegetation indices, finding that the coefficients of determination for the estimation models throughout the rice growth period ranged from 0.59 to 0.75, but were only around 0.2 during the grain-filling period [22]. These findings collectively highlight the inherent limitations of spectral-only approaches for mature rice biomass estimation.

To address the limitations of spectral indices, researchers have integrated auxiliary phenotypic traits (e.g., texture features, plant height, and canopy structure) with spectral data to enhance biomass estimation accuracy. This multi-modal fusion strategy effectively mitigates spectral saturation effects and improves model robustness across crop growth stages [23,24,25,26,27,28]. For example, Wan et al. combined UAV-derived spectral indices with plant height and canopy coverage parameters, achieving interannual prediction consistency with *R*^2^ values of 0.83–0.85 for rice yield models [29]. Additionally, Fu constructed a rice biomass estimation model by fusing color indices and texture features during the tillering, panicle initiation, and heading stages. They found that this approach outperformed single-index models, with the highest model determination coefficient reaching 0.92. However, while the canopy spectral reflectance signal of rice is highly sensitive to biomass changes during these stages, the study did not investigate the estimation accuracy of rice biomass in the later maturity stage, when the grains gradually mature and the leaves begin to yellow and senesce [30]. Similarly, studies on wheat biomass estimation have yielded comparable conclusions. For example, Zheng utilized a wheat biomass estimation model that integrates spectral and texture features, demonstrating that feature fusion enhances the estimation accuracy compared to using a single feature [31]. Dai M et al. further quantified the benefits of multi-feature integration: while late-growth-stage models using single spectral indices showed limited explanatory power (*R*^2^ = 0.464), incorporating texture indices increased *R*^2^ to 0.515 [32]. Collectively, these studies confirm two critical observations: multi-feature fusion significantly improves early-growth biomass estimation and yield prediction; and accuracy gains diminish substantially in late growth stages, particularly at maturity. This persistent limitation underscores the need for novel feature combinations or methodological innovations to overcome saturation challenges in crop senescence phases.

To address these limitations and holistically characterize mature rice biomass, focusing on mature rice, we start with the definition of above-ground biomass and integrate RGB vegetation indices from rice canopy imagery with growth parameters, such as rice plant height and moisture content, to construct a biomass estimation model. The RGB vegetation indices primarily provide spectral information on the growth status of rice, while the growth parameters offer direct insights into the growth conditions. The model is then applied to estimate the biomass of mature rice in the experimental area. An analysis of biomass distribution within the experimental zone is conducted to further validate the model’s reliability. This research aims to provide new and improved references for the accurate estimation of biomass in mature rice. It also offers a more comprehensive scientific basis for monitoring rice growth and assessing yield.

## 2. Materials and Methods

### 2.1. Overview of the Experimental Area

The experimental area is located at the Nanping Agricultural Machinery Service Professional Cooperative in Xiangxiang City, Hunan Province (112.5° E, 27.8° N). Xiangxiang City features a mid-subtropical monsoon humid climate, characterized by distinct seasons, abundant rainfall, and a frost-free period of 265–275 days. The rice planting area in the experimental site is approximately 30 hm^2^. The rice variety is Zhuliangyou 819, planted at a density of 15 cm between rows and 10 cm between plants, with natural irrigation. Sowing occurs in early April, and harvesting takes place in mid-to-late July each year. During the rice growing period, the average temperature is 24.5 °C, with extreme maximum and minimum temperatures of 42 °C and 7 °C, respectively, and an average monthly precipitation of 113.6 mm.

**Figure 1:**
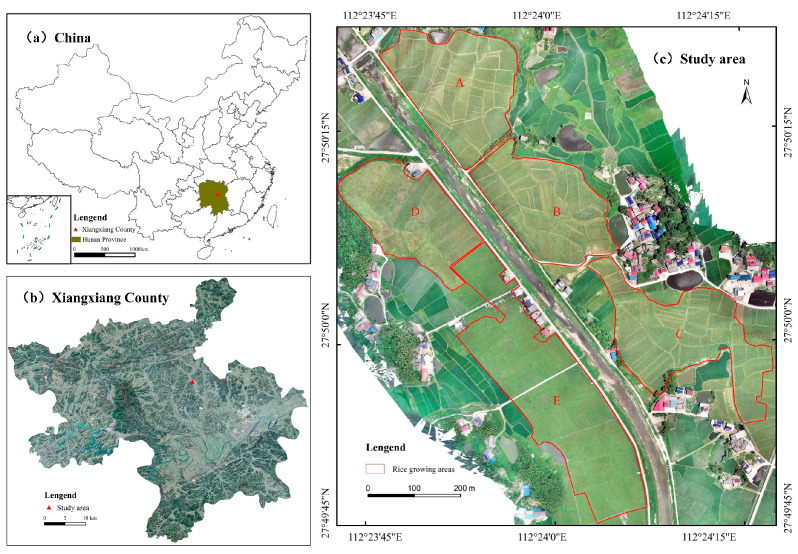
Overview of the experimental area. (**a**) China, (**b**) Xiangxiang County, (**c**) study area.

This experiment was conducted on 14 July 2023, and 20 July 2024, when the rice was in the mature stage. Due to the large rice planting area and the extended harvesting period, the experimental area was divided into five zones (A–E) based on geographical location and harvesting schedule to specifically analyze rice biomass estimation. The overview of the experimental area and its division are presented in Figure 1.

### 2.2. Data Collection and Processing

Based on the growth condition of rice, unmanned aerial vehicle (UAV) remote sensing data and ground data in the experimental site were simultaneously collected on 14 July 2023 and 20 July 2024. At this time, the rice in the experimental area was generally 25–35 days after flowering and had entered the mature stage. The UAV remote sensing data included RGB and multispectral data, while the ground data included rice plant height, moisture content, and biomass information. Multiple sampling points were randomly selected based on geographic location, and a 1 m × 1 m rectangular sampling area was established around each sampling point. Within each sampling area, five rice plants were selected, and their average values were taken as the sample data for that sampling point, resulting in a total of 75 effective samples collected on-site.

1. Ground Data Collection

**Figure 2:**
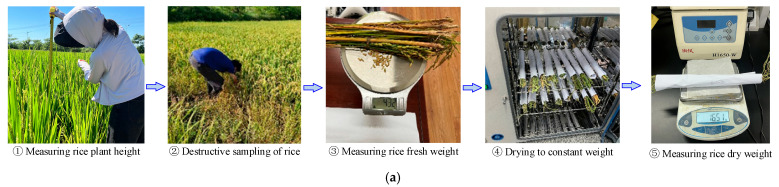

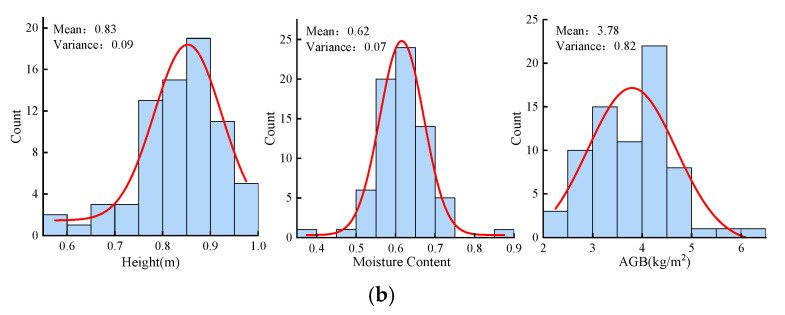
Ground data collection, including (**a**) the collection process and (**b**) the distribution of data.

The primary processes for ground data collection were as follows:

(1) Measurement of rice plant height: The distance from the base to the top of the canopy of rice plants was measured using a tape measure while the plants were in their natural growth state, and the height was recorded (unit: cm).
(2) Collection of fresh weight: Destructive sampling was conducted, where samples were bagged and labeled for identification, followed by weighing in the laboratory to obtain the fresh weight of the rice (unit: kg).
(3) Collection of dry weight: The rice samples were placed in a laboratory oven, heated at 105 °C for 30 min, and then dried at 75 °C for approximately 48 h until a constant weight was achieved. The final recorded weights were averaged to represent the dry weight (in kg) of the rice plant samples from the experimental area. This average dry weight was then converted to above-ground dry biomass per square meter based on the planting density of the rice crop (unit: kg/m^2^).
(4) Determination of moisture content: The moisture content of the rice plants was calculated as the difference between the fresh weight and dry weight of the rice plants divided by the fresh weight, as illustrated in Figure 2a.

Finally, through the aforementioned ground data collection process, data on rice plant height, moisture content (MC), and above-ground biomass (AGB) were obtained. Statistical analysis indicated that the data collection conformed to a normal distribution, suggesting a certain degree of reliability in the field sample data collection, as shown in Figure 2b.

2. Drone Remote Sensing Data Acquisition and Analysis

Simultaneously with the ground data collection, we used the DJI Phantom 4 UAV (Shenzhen Dajiang Innovation Technology Co., Ltd., Shenzhen, Guangdong, China) to acquire RGB data from the experimental area. The UAV is equipped with a 1/2.3-inch CMOS image sensor, has 12.4 million effective pixels, and features a compact size and lightweight design, with a maximum flight duration of approximately 30 min. The flight parameters were set as follows: altitude at 100 m, speed at 7 m/s, forward overlap at 80%, lateral overlap at 70%, and flight angles at 0° and −45°. Additionally, we used the DJI Matrice M300 RTK UAV (Shenzhen Dajiang Innovation Technology Co., Ltd., Shenzhen, China), equipped with the MS600 Pro (Changguang Yuchen Information Technology and Equipment Co., Ltd., Qingdao, China) six-channel multispectral sensor, to gather multispectral data. The multispectral camera includes six independent spectral channels, with central wavelengths of blue (450 nm), green (555 nm), red (660 nm), two red edges (720 nm, 750 nm), and near-infrared (840 nm). The flight altitude was set at 80 m, with both forward and lateral overlaps at 80% and 70%, respectively, at a flight angle of 0°.

A total of 1754 nadir RGB images, 5769 45° oblique RGB images, and 4762 multispectral images per band were collected. The raw RGB and multispectral images collected by UAV were processed for stitching and correction using Pix4D Mapper (Version 4.4.12) and Yusense Map (Version 2.2.3) software. Subsequently, the UAV imagery was cropped according to the location of the sampling areas, and the average reflectance for each sampling area was calculated to represent the spectral reflectance at each sampling point. This process ultimately yielded spectral reflectance data across different wavelengths.

3. Construction of Vegetation Indices

Vegetation indices are crucial parameters for characterizing crop growth information through the combination of spectral data. Based on the estimation requirements for rice biomass, 11 commonly used RGB vegetation indices were selected as indicators for constructing the rice biomass model during the maturity stage [33,34,35,36,37]. Additionally, drawing on relevant literature [38,39,40,41], 6 multispectral indices with high correlation with measured moisture content were chosen as indicators for regional moisture content estimation. Table 1 lists the 11 RGB vegetation indices and 6 multispectral indices, along with their respective formulas.

### 2.3. Technical Approach

This paper presents a biomass estimation model for mature rice. The model integrates RGB vegetation indices and growth parameters and is applied to the entire experimental area to derive the spatial distribution of mature rice biomass. The study is divided into three main sections:

1. Data Collection and Preprocessing: This section includes the following experimental data: (1) UAV remote sensing data: comprising RGB vegetation indices and multispectral data; (2) ground sampling data: including rice plant height, moisture content, and biomass data.
2. Development of the Biomass Estimation Model for Mature Rice: This section consists of two main components: (1) selection of key variables: this involves identifying the key variables most relevant to biomass estimation; (2) model formulation: constructing a biomass estimation model based on the selected key variables.
3. Application of the Model: In this section, the optimal biomass estimation model for mature rice is used to estimate biomass and analyze influencing factors within the experimental area.

**Figure 3:**
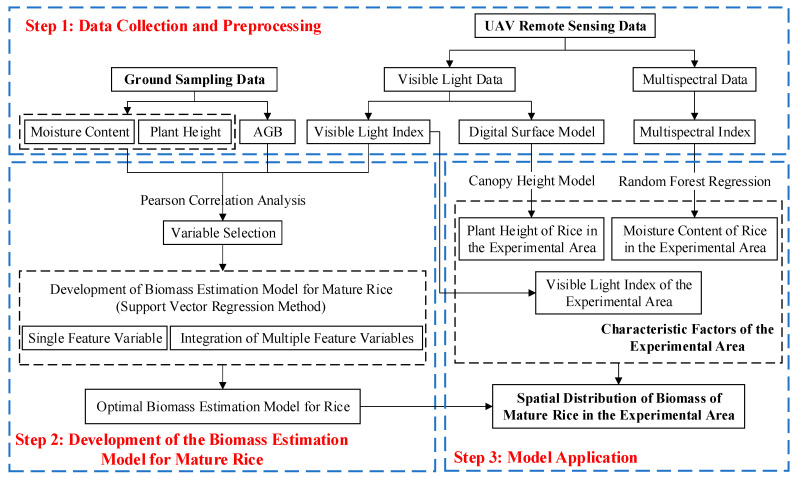
Research flowchart.

The specific technical workflow is illustrated in Figure 3, which provides a visual overview of the steps involved in the study.

### 2.4. Data Training and Accuracy Assessment

1. Model Training

Support vector regression (SVR) effectively captures nonlinear relationships between variables by identifying an optimal hyperplane that minimizes deviation from actual values within a specified tolerance [42,43]. This method is particularly adept at handling high-dimensional data and exhibits superior generalization capabilities. However, its performance critically depends on empirical determination of hyperparameters and kernel functions [44]. Consequently, we implemented SVR with parameters derived from experimental data distribution analysis to estimate rice biomass. To ensure comparability across different experiments, all trials adopted identical model settings, including a penalty coefficient *C* = 6.5, a radial basis function (RBF) kernel, and a tolerance *ϵ* = 0.5. Parameter optimization was guided by data characteristics and iterative model evaluation. Through five-fold cross-validation, this combination achieved maximal estimation accuracy while preserving model generalizability.

The experimental dataset contained RGB vegetation indices of the mature rice canopy, plant height, moisture content, and biomass measurements. Data were partitioned into training–validation (85%) and independent test (15%) sets, with consistent splitting ratios applied to all experiments. Model development utilized the training–validation set with cross-validation, while final evaluation was performed on the held-out test set.

2. Precision Evaluation

The accuracy of the model’s estimated rice biomass compared to the actual measured biomass was quantitatively evaluated using linear regression analysis. The coefficient of determination (*R*^2^) and the root mean square error (*RMSE*) were used as evaluation metrics, with specific formulas as shown in Equations (1) and (2). A 1:1 line (identity line) was used to compare the consistency between the estimated and actual values of rice biomass across different models. When the model’s estimation closely matches the 1:1 line, it indicates a high degree of consistency between the model’s estimated results and the actual measured results, thereby proving the reliability and validity of the model.

(1) $$R^{2}=1-\frac{\sum_{i=1}^{n}{\left(y_{i}-{\hat{y}}_{i}\right)}^{2}}{\sum_{i=1}^{n}{\left(y_{i}-{\bar{y}}_{i}\right)}^{2}}$$

(2) $$RMSE=\sqrt{\frac{1}{n}\sum_{i=1}^{n}{\left(y_{i}-{\hat{y}}_{i}\right)}^{2}}$$

Here, *n* is the number of rice plants; *y_i_*, ${\bar{y}}_{i}$, and ${\hat{y}}_{i}$ are the actual measured biomass, the average value, and the model-predicted value of rice biomass, respectively. The coefficient of determination (*R*^2^) indicates the degree of fit between the estimated values and the actual measured values, while the root mean square error (*RMSE*) measures the error between the estimated values and the actual measured values. Higher *R*^2^ and lower *RMSE* values indicate a better match fit between the estimated and actual values, suggesting more accurate estimation results.

## 3. Results

### 3.1. Estimation of Biomass of Mature Rice Based on Single Feature Variables

1. Correlation Analysis

**Figure 4:**
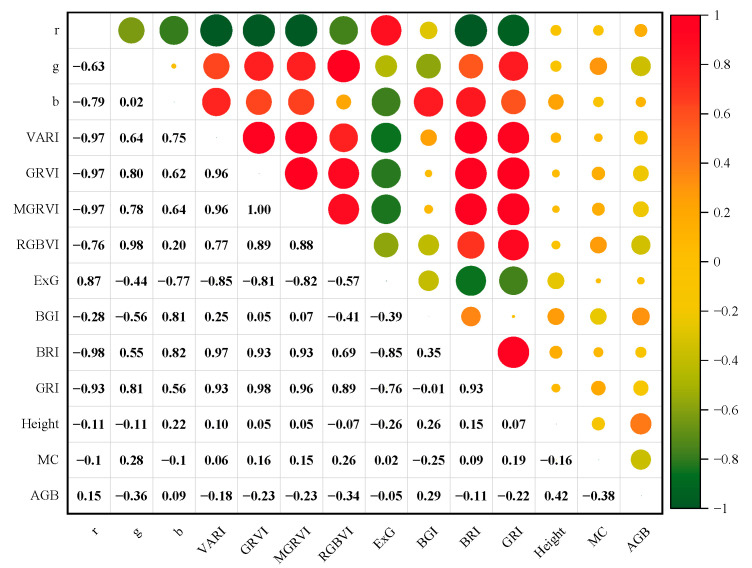
Analysis of the correlation between single feature variables and biomass.

To explore the relationship between the RGB vegetation indices of the rice canopy at maturity and the rice growth parameters with biomass, Pearson correlation analysis was initially used to assess the correlation between the 11 RGB vegetation indices selected in this study and rice biomass (AGB). Additionally, the correlation between rice plant height (Height), moisture content (MC), and rice biomass was also evaluated, as shown in Figure 4. Based on the correlation between each feature variable and biomass, the following key observations can be made:

During the mature stage, the correlation between the RGB vegetation indices of the rice canopy and biomass is generally weak. Among them, the indices with higher correlation are g and RGBVI, with correlation coefficients of −0.36 and −0.34, respectively. Some indices exhibit a correlation around 0.1 with biomass, suggesting a weak relationship. The correlation coefficients between rice plant height (Height), moisture content (MC), and biomass are 0.42 and −0.38, respectively, which are both higher than the correlation between the RGB vegetation indices and biomass.

2. Estimation of Biomass of Mature Rice Based on Single Feature Variables

**Figure 5:**
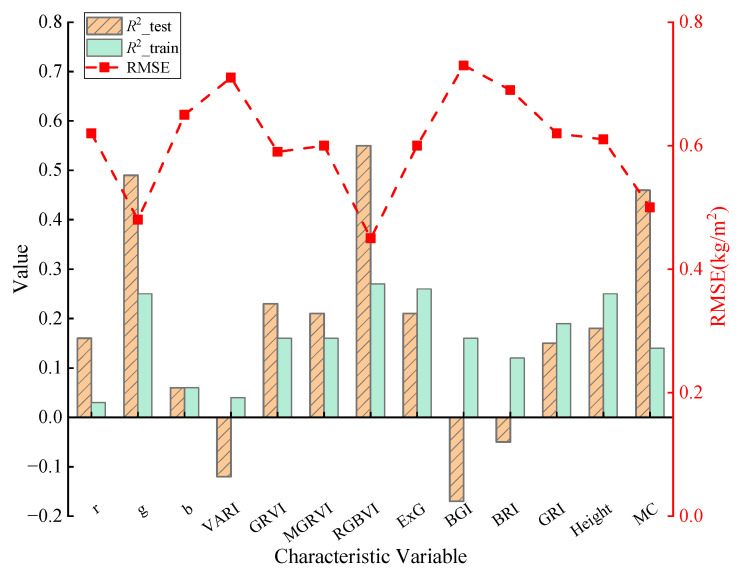
Precision of biomass estimation based on single feature variables.

Based on the results of the Pearson correlation analysis, it was found that the linear relationship between a single RGB vegetation index or growth parameter and rice biomass during the mature stage is generally weak. To further explore whether there is a more complex relationship between these variables, the eleven RGB indices and two growth parameters (plant height and moisture content) were used as independent variables. The support vector regression (SVR) method was used to construct the biomass estimation model for mature rice. The precision of the model on the training set and the test set was evaluated using the coefficient of determination (*R*^2^) for the training set (*R*^2^*_train_*) and the test set (*R*^2^*_test_*), respectively, as shown in Figure 5.

The modeling results are summarized as follows:

(1) The precision of the training set for biomass estimation using only a single RGB vegetation index is generally low, with the coefficient of determination (*R*^2^*_train_*) ranging from 0 to 0.3, indicating a poor fit to the training data. Among these, the models constructed using the green band normalized value (g), red–green–blue vegetation index (RGBVI), and excess green vegetation index (ExG) exhibit relatively higher *R*^2^*_train_* values of 0.25, 0.27, and 0.26, respectively.
(2) On the model test dataset, the model constructed based on the visible light atmospherically resistant vegetation index (VARI), blue–green ratio index (BGI), and blue–red ratio index (BRI) exhibited a negative determination coefficient on the test set, indicating that the model’s predictive ability was weaker than that of a simple mean prediction [45]. Therefore, these three variables were removed through feature selection in subsequent experiments. The *R*^2^*_test_* of the other feature variables is greater than 0, with the model based on the red–green–blue vegetation index (RGBVI) performing the best, achieving an *R*^2^*_test_* of 0.55 and an *RMSE* of 0.45 kg/m^2^.
(3) Among the growth parameters, the precision of the training set (*R*^2^*_train_*) for biomass estimation using plant height (Height) and the precision of the test set (*R*^2^*_test_*) for biomass estimation using moisture content (MC) are both relatively high, with coefficients of determination of 0.25 and 0.46, respectively.

It can be concluded that using only RGB indices or growth parameters as single feature variables may not sufficiently capture the variations in the biomass of mature rice. The precision of the biomass estimation models constructed using these single feature variables is generally low. Therefore, we selected eight RGB vegetation indices and two growth parameters as input factors and integrated multiple types of feature variables for modeling analysis.

### 3.2. Development of Biomass Estimation Models Based on Multi-Feature Fusion

The biomass estimation results for mature rice using single RGB vegetation indices or growth parameters as independent variables showed limited accuracy. Therefore, we integrated multiple types of feature variables to construct the modeling analysis.

#### 3.2.1. The Impact of RGB Vegetation Indices Screening on Rice Biomass Estimation

1. Screening of RGB Vegetation Indices

Given the biomass estimation results using single feature variables, a total of eight RGB vegetation indices were selected. The coefficient of determination (*R*^2^*_test_*) of the biomass estimation model for single RGB vegetation indices on the test set was used as the criterion to classify the RGB vegetation indices into five types for indicator screening. The specific classification criteria are shown in Table 2.

2. The Impact of RGB Vegetation Indices Screening on Rice Biomass Estimation

**Figure 6:**
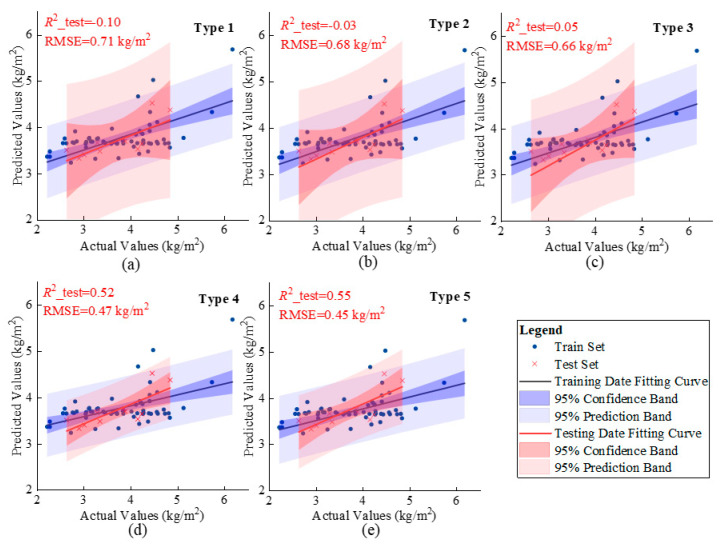
This is a graph showing the relationship between estimated and actual biomass values based on different categories of RGB vegetation indices, where (**a**) represents Type 1, (**b**) represents Type 2, (**c**) represents Type 3, (**d**) represents Type 4, and (**e**) represents Type 5.

To explore the impact of the screening of RGB vegetation indices on rice biomass estimation, mature rice biomass estimation models were constructed using different categories of RGB vegetation indices as input factors. The estimation results show clear differences in the relationships between RGB vegetation indices divided by different classification standards and biomass, as depicted in Figure 6.

Specifically, when the classification standards were set as Type 1 and Type 2, the determination coefficient of the biomass estimation model on the test set was less than 0, and the scatter distribution of the estimated and measured biomass values was relatively dispersed. When the classification standard was set as Type 3, the determination coefficient of the model test set was greater than 0, but the model precision stayed relatively low, with a slightly more concentrated scatter distribution, yet the dispersion degree was still large. Therefore, the estimation precision of Type 1–3 models persisted at a low level, indicating that RGB vegetation indices with low correlation contributed little to biomass estimation and significantly impacted model precision. Moreover, incorporating numerous low-correlation feature variables considerably affected the model estimation precision.

When the classification standard was set as Type 4 or Type 5, the determination coefficient of the constructed biomass estimation model on the test set was significantly improved, reaching 0.52 and 0.55, respectively. The scatter distribution of the estimated biomass values and measured values became more concentrated, clustering around the fitted line and aligning with it. Compared with the fitting results of other RGB vegetation indices, the confidence interval and prediction interval were markedly narrower, indicating reduced model uncertainty and enhanced prediction accuracy. This implies that RGB vegetation indices with higher correlation can markedly enhance the model estimation precision.

The results suggest that when multiple RGB vegetation indices are used simultaneously as model factors for rice biomass estimation, the screening of these indices is a crucial factor influencing the estimation precision.

#### 3.2.2. Development of Rice Biomass Estimation Models Integrated with Growth Parameters

Based on the impact of RGB vegetation indices screening on biomass estimation, RGB vegetation indices with high correlation can significantly enhance the accuracy of biomass estimation. However, the estimation accuracy remains comparatively low. To further improve the estimation accuracy of mature rice biomass from multiple dimensions, modeling analysis was performed by incorporating growth parameters, such as rice plant height and moisture content, alongside RGB vegetation indices.

**Figure 7:**
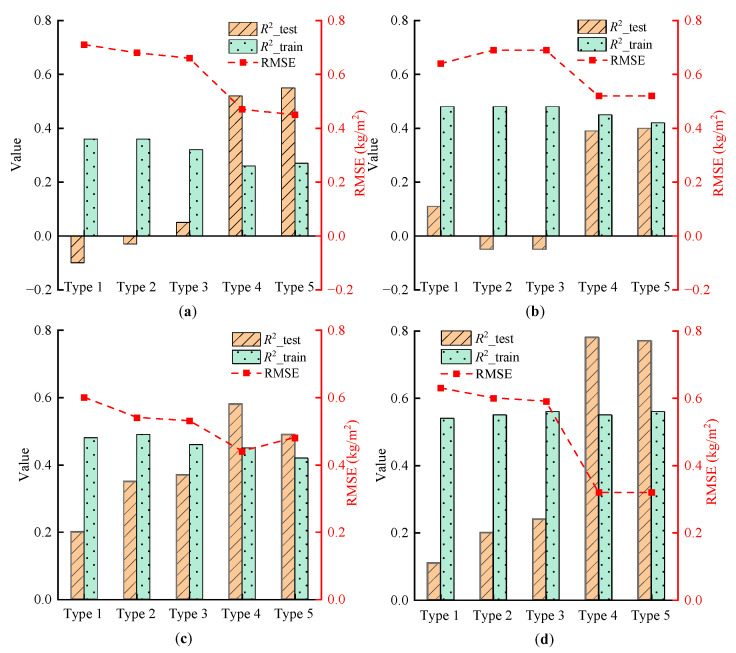
Accuracy of biomass estimation models based on multi-feature fusion, where (**a**) models are built based on various RGB vegetation indices, (**b**) adds rice plant height to the models based on various RGB vegetation indices, (**c**) adds rice moisture content to the models based on various RGB vegetation indices, and (**d**) simultaneously adds rice plant height and moisture content to the models based on various RGB vegetation indices.

Figure 7 illustrates the accuracy of biomass estimation models integrating different categories of RGB vegetation indices with growth parameters. The observations are as follows:

1. When only RGB vegetation indices are used as input factors for constructing the biomass estimation model of mature rice, the coefficient of determination (*R*^2^) for the test set of Type 1 and Type 2 indices is less than 0, and for Type 3 indices, the *R*^2^ is less than 0.1, indicating poor model fit. In contrast, for Type 4 and Type 5 indices, the *R*^2^ is approximately 0.5, suggesting that the screening of RGB vegetation indices significantly impacts model accuracy.
2. Incorporating rice plant height into the RGB vegetation indices results in a decrease in the *R*^2^ for the test set of the biomass estimation model, with some values still lodging below 0 and the root mean square error (*RMSE*) remaining relatively high. Conversely, adding only rice moisture content significantly improves the *R*^2^ for the test set, with the most accurate model achieving an *R*^2^ of 0.58 and an *RMSE* of 0.44 kg/m^2^. When both rice plant height and moisture content are included, the model’s fit to the training set is improved. Notably, the biomass estimation model for mature rice using Type 4 RGB vegetation indices achieves the highest precision, with an *R*^2^ and *RMSE* of 0.78 and 0.32 kg/m^2^, respectively.
3. The screening of RGB vegetation indices and the integration of growth parameters are crucial for enhancing the accuracy of biomass estimation for mature rice. Therefore, using the green band normalized value (g), red–green–blue vegetation index (RGBVI), rice plant height, and rice moisture content as input variables results in the optimal model for estimating mature rice biomass. This finding indicates that the integration of growth parameters can compensate for the limitations of RGB vegetation indices, thereby improving the model’s fit to the data.

### 3.3. Acquisition of Growth Parameters of Mature Rice in the Experimental Area

To explore the application of the model in estimating rice biomass over a larger area, it is necessary to obtain two RGB vegetation indices of the mature rice canopy in the experimental area, namely the green band normalized value (g) and the red–green–blue vegetation index (RGBVI), as well as two growth parameters: plant height and moisture content. The RGB vegetation indices data can be directly extracted from the RGB images of the experimental area. However, rice plant height and moisture content data cannot be directly obtained. Therefore, following previous research methods, the canopy height model (CHM) is employed to extract the plant height of mature rice in the experimental area, and the moisture content of rice in the experimental area is estimated using multispectral imagery and the random forest regression method.

1. Rice Plant Height

In this experiment, UAV remote sensing data were collected both prior to rice planting, when the paddy field was bare, and during the mature stage of rice. The collected data were processed to generate corresponding digital surface models (DSMs). These DSMs represent the ground height of the paddy field and the height of the mature rice canopy. The plant height data of the mature rice were calculated using the canopy height model (CHM), which is derived by subtracting the DSM of the bare period from the DSM of the mature period, as shown in Equation (3). Finally, the plant height data obtained from UAV remote sensing were fitted and analyzed in conjunction with the measured plant height.

(3) $$CHM=DSM_{\mathrm{maturity}}-DSM_{\mathrm{bare}\;\mathrm{ground}}$$

where *CHM* is the canopy height model of rice at maturity, and *DSM*_maturity_ and *DSM*_bare ground_ are the digital surface models when rice is at maturity and when rice is bare ground before sowing, respectively.

**Figure 8:**
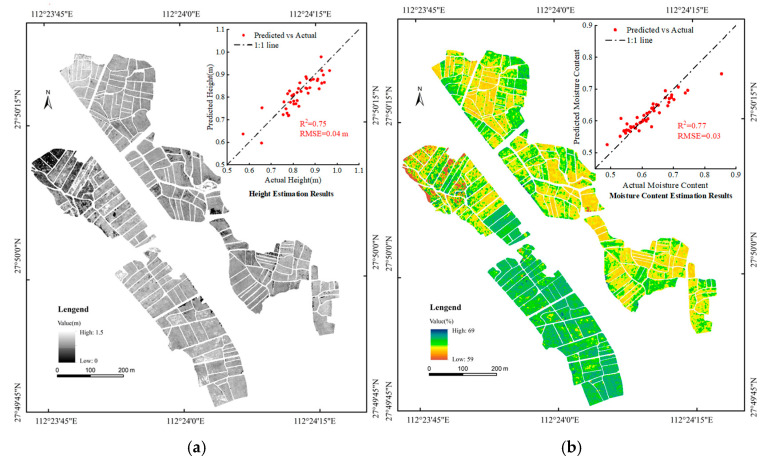
Extraction of rice growth parameters in the experimental area, including (**a**) CHM-based rice plant height extraction and (**b**) RFR-based rice moisture content acquisition.

The results indicate that the rice plant height extracted based on the canopy height model closely matches the actual measured plant height, with an *R*^2^ of 0.75 and an *RMSE* of 0.04 m. Figure 8a illustrates the distribution of plant height of mature rice in the experimental area.

2. Rice Moisture Content

Rice moisture content (MC) is a critical factor influencing its growth and biomass. Accurate estimation models for monitoring rice moisture content can be developed using multispectral indices derived from high-resolution multispectral images captured by un-manned aerial vehicles (UAVs) [46,47,48]. In this experiment, six multispectral vegetation indices with high correlation to the actual measured moisture content, such as the difference vegetation index (DVI), and three multispectral band data, namely the red light band reflectance, red edge band reflectance, and near-infrared band reflectance, were extracted from the multispectral images of the experimental area. These indices and band data effectively capture the spectral characteristics of the rice canopy, enhancing the accuracy of moisture content estimation. Subsequently, a random forest regression (RFR) model was employed to establish the relationship between multispectral indices and moisture content, with the following parameter settings: 1000 decision trees, maximum depth set to None, minimum samples per split set to 2, and other parameters using the default settings of the Python (Version 3.10.14) scikit-learn (Version 1.4.2) library’s random forest regression. The results indicated that the estimation of moisture content for mature rice using multispectral data and the random forest regression method was highly accurate, with a model coefficient of determination (*R*^2^) of 0.77. The scatter plot showed minimal deviation from the 1:1 line, indicating a good fit. The distribution of moisture content for mature rice in the experimental area is illustrated in Figure 8b.

### 3.4. Biomass Estimation of Mature Rice in the Experimental Area

**Figure 9:**
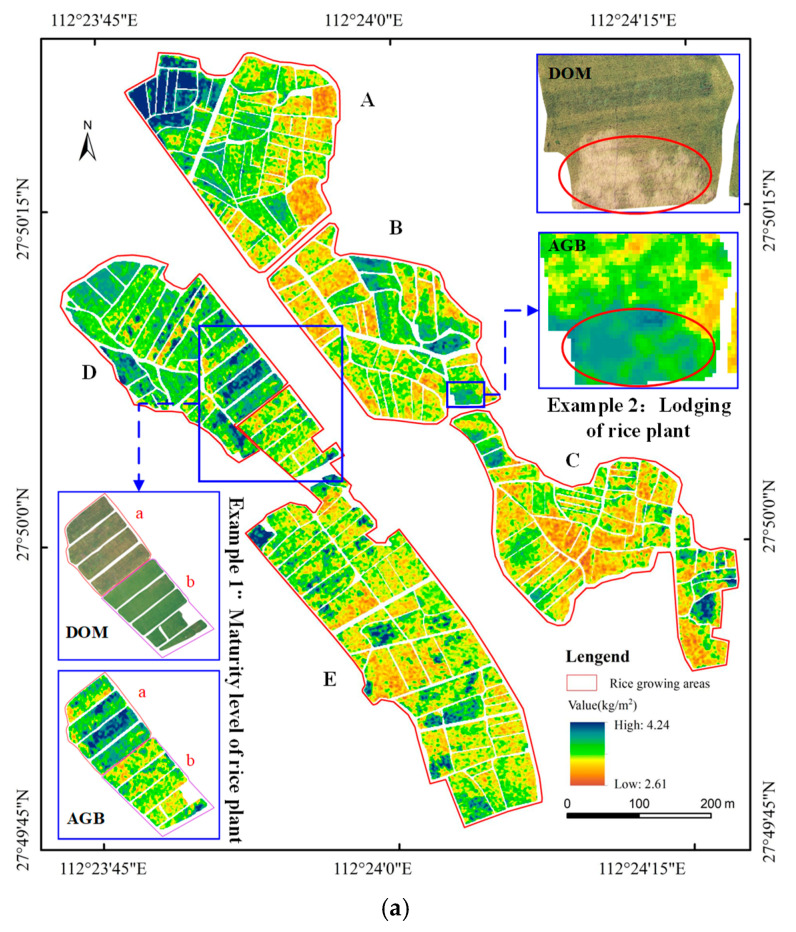

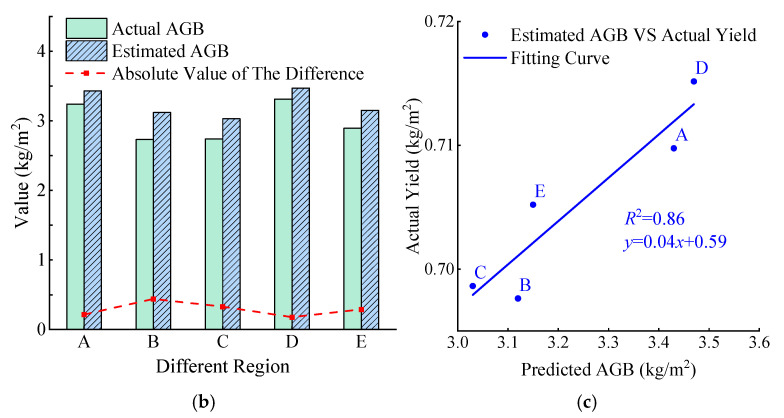
Analysis of the estimated biomass results of rice in the experimental area, mainly including (**a**) spatial distribution of rice biomass in the experimental area, as well as cases of rice with different degrees of maturity and lodging; (**b**) comparison between estimated and measured biomass values; (**c**) fitting results of estimated biomass and actual yield.

Using growth parameters obtained from the experimental area and combining them with two RGB vegetation indices (g, RGBVI), a rice biomass estimation model for the maturity stage was constructed to estimate the biomass of rice at this stage in the experimental area. The distribution of rice biomass at maturity was obtained, and a statistical analysis was conducted using the measured biomass and yield. The statistical results indicate significant differences in biomass across different areas, primarily characterized as follows:

1. Based on the statistical analysis of the estimated and measured rice biomass values at maturity in each area (Figure 9b), there is a consistent relationship between the estimated and measured values. The estimated values are generally higher than the measured values, but the differences are small, ranging from 0.15 to 0.39 kg/m^2^. Overall, the model performs well; however, the difference in Area B is relatively high, indicating a larger estimation deviation. Regarding the biomass distribution in the experimental area, the overall biomass level is highest in Areas D and A, while it is relatively lower in Areas E, B, and C. Moreover, variations in biomass were observed within each individual area.
2. A fitting analysis was conducted between the estimated biomass and the measured yield in each area of the experimental region (Figure 9c). The coefficient of determination for the fit is 0.86 and the fitting function is *y* = 0.04*x* + 0.59. The fitting results show a strong linear correlation between yield and biomass. The yield in each area is consistent with biomass, meaning that areas with higher biomass values also have higher yields.

These results indicate that the model has a certain degree of reliability for large-area rice biomass estimation at the maturity stage, but some factors still affect the estimation accuracy. To further explore the influencing factors of rice biomass estimation at maturity, we conducted a specific analysis based on the rice biomass situation and growth conditions during data collection, focusing on two aspects: the maturity degree and lodging condition of rice.

1. The Impact of Rice Maturity on Biomass Estimation

First, the maturity degree of rice was assessed using visible imagery features. As shown in Example 1 in Figure 9a, the maturity of rice in Region a is greater than that in Region b, and the biomass estimation result in Region a is also higher than that in Region b. Second, the maturity of rice is determined by the harvesting time. For example, the harvesting time for Region D was 18 July 2023 and for Region E was 29 July 2023, while the UAV data collection occurred on 14 July 2023. This indicates that Region D was more mature than Region E. The biomass estimation results indicate that the biomass in Region D had a higher maturity than Region E. Therefore, a higher maturity of rice corresponds to a higher biomass value, suggesting that rice maturity is one of the main factors affecting the model’s estimation accuracy.

2. The Impact of Rice Lodging on Biomass Estimation

Lodging of rice is a common phenomenon in paddy fields due to various natural factors. By visually interpreting the orthorectified images (DOM) of rice in the experimental area, obtained via UAV remote sensing, it is observed that the area shown in Example 2 in Figure 9a is a large lodging area in Region B. An analysis of the rice biomass distribution revealed significant differences in the estimated biomass values between lodging areas and other regions. Specifically, the estimated biomass values in the lodging areas are much higher than those in other areas. This suggests that lodging areas have a significant impact on the rice biomass estimation and are also key factors affecting yield estimation accuracy.

## 4. Discussion

This study proposes an estimation model that integrates RGB vegetation indices with growth parameters (plant height and moisture content) to address the challenges of leaf yellowing, drying, and spectral index saturation in estimating the biomass of mature rice.

The spectral indices of mature rice tend to saturate due to leaf yellowing and drying, resulting in a weak correlation between single spectral indices and biomass (with the maximum correlation coefficient being only −0.36) and low modeling accuracy (the determination coefficient of the training set ranges from 0 to 0.3, and the highest for the test set is 0.55). This result is consistent with previous studies [49,50,51], indicating the limitations of traditional spectral methods in estimating biomass during the maturity stage.

To address the issue of the limited contribution from single RGB vegetation indices, this study screened RGB vegetation indices with higher modeling accuracy based on single indices and combined them with growth parameters, further improving model accuracy. It was found that RGB vegetation indices with low modeling accuracy contribute less to biomass estimation, and adding too many low-accuracy indices can degrade model performance. Therefore, feature screening is one of the key factors affecting estimation accuracy when constructing multi-feature models. Additionally, plant height and moisture content were introduced as growth parameters. Plant height, as an important morphological indicator of rice growth height and biomass accumulation, directly reflects the growth condition of rice; moisture content reflects the physiological and health status of rice [52]. After integrating growth parameters, the model’s ability to fit the data was significantly enhanced, indicating that multi-feature fusion can effectively alleviate the problem of spectral index saturation and enhance the monitoring ability of crop growth changes.

When applying the model to estimate the biomass of rice in the experimental area, it was found that the higher the maturity of rice, the greater the biomass value, confirming the positive correlation between maturity and biomass. Meanwhile, rice lodging can reduce the recognition accuracy of plant height and moisture content, thereby affecting the estimation results. Therefore, the impact of lodging should be considered in the estimation during the maturity stage.

There are still some limitations in this study, mainly reflected in the following aspects: First, the data only cover rice in the mature stage and do not take into account the impact of conditions in other growth stages on biomass. Second, the traditional support vector regression method was directly used to construct the model without any improvement in the modeling method. Third, the small sample size may affect the stability and adaptability of the model. Future research can expand the sample size and optimize the modeling method to enhance the robustness and universality of the model.

## 5. Conclusions

This paper constructs a biomass estimation model for mature rice by integrating RGB vegetation indices of the rice canopy, obtained via unmanned aerial vehicles (UAVs), with growth parameters such as plant height and moisture content measured on the ground, based on the support vector regression method. The model was applied to estimate the biomass of mature rice in the experimental area. The results of this study show the following:

1. Based on the feature screening strategy, eight RGB vegetation indices including the green band normalized value (g) and the red–green–blue vegetation index (RGBVI) were selected, combined with plant height and moisture content to form a multi-feature set. In single-variable models, the g and RGBVI indices exhibited *R*^2^ values of 0.49 and 0.55 on the test set, with corresponding *RMSE* values of 0.48 kg/m^2^ and 0.45 kg/m^2^, respectively, demonstrating their relatively good ability to characterize biomass.
2. The biomass estimation model combining RGB vegetation indices (g, RGBVI) and growth parameters (plant height, moisture content) achieved an *R*^2^ value of 0.78 on the test set, with a reduced *RMSE* of 0.32 kg/m^2^. This validates the effectiveness of the multi-feature fusion method in alleviating the saturation problem of spectral indices and enhancing the model’s predictive capability.
3. Application of the model in the experimental area shows that the absolute difference between the estimated biomass values and the measured values is within 0.15–0.39 kg/m^2^, and a significant linear relationship with the measured yield (*y* = 0.04*x* + 0.59, *R*^2^ = 0.86) is observed, indicating the model’s reliability for large-area biomass estimation of mature rice. However, the degree of rice maturity and lodging phenomena can lead to a decrease in the accuracy of model application.

In summary, the proposed method offers a novel approach for the rapid and efficient estimation of biomass in mature rice, providing a methodological reference for rice growth monitoring and yield assessment. Future research can further enhance the universality and reliability of the model by increasing the sample size, improving modeling methods, and enhancing the accuracy of growth parameter extraction.

## Figures and Tables

**Table 1:** The vegetation indices used in this study.

Number	Vegetation Index	Calculation Formula	Number	Vegetation Index	Calculation Formula
RGB vegetation indices					
1	r	R/(R+G+B)	7	BGI	B/G
2	g	G/(R+G+B)	8	BRI	B/R
3	b	B/(R+G+B)	9	GRI	G/R
4	VARI	(G−R)/(R+G+B)	10	ExG	2×G−R−B
5	GRVI	(G−R)/(G+R)	11	RGBVI	$\left(G^{2}-R\times B\right)/\left(G^{2}+R\times B\right)$
6	MGRVI	$\left(G^{2}-R^{2}\right)/\left(G^{2}+R^{2}\right)$	/	/	/
multispectral Indices					
1	RVI	$NIR/R^{*}$	4	OSAVI	$\left(NIR-R^{*}\right)/\left(NIR+R^{*}+0.16\right)$
2	DVI	$NIR-R^{*}$	5	EVI2	$2.5\times (NIR-R^{*})/\left(NIR+2.4R^{*}+1\right)$
3	NDVI	$\left(NIR-R^{*}\right)/\left(NIR+R^{*}\right)$	6	RESAVI	1.5×(NIR−RE)/(NIR+RE+0.5)

Note: r stands for red light normalized value, g for green light normalized value, b for blue light normalized value. VARI denotes the visible atmospherically resistant index, GRVI the green–red vegetation index, MGRVI the modified green–red vegetation index, BGI the blue–green index, BRI the blue–red index, GRI the green–red index, ExG the excess green index, and RGBVI the red–green–blue vegetation index. RVI denotes the ratio vegetation index, DVI the difference vegetation index, NDVI the normalized difference vegetation index, OSAVI the optimized soil adjusted vegetation index, EVI2 the two-band enhanced vegetation index, and RESAVI the red edge soil adjusted vegetation index. R represents the red light reflectance, G represents the green light reflectance, and B represents the blue light reflectance in RGB images. NIR represents the near-infrared reflectance, R* represents the red light reflectance, and RE represents the red edge reflectance in multispectral images.

**Table 2:** Classification of RGB vegetation indices.

Type	Criteria	Selection of RGB Vegetation Indices
Type 1	*R*^2^*_test_* > 0	r, g, b, GRVI, MGRVI, RGBVI, ExG, GRI
Type 2	*R*^2^*_test_* > 0.1	r, g, GRVI, MGRVI, RGBVI, ExG, GRI
Type 3	*R*^2^*_test_* > 0.2	g, GRVI, MGRVI, RGBVI, ExG
Type 4	*R*^2^*_test_* > 0.4	g, RGBVI
Type 5	*R*^2^*_test_* > 0.5	RGBVI

Note: The *R*^2^*_test_* in the table represents the test precision of the biomass estimation model based on single feature variables.

## Data Availability

Data are contained within the article.

## Abbreviations

The following abbreviations are used in this manuscript:

AGB	above-ground biomass
UAV	unmanned aerial vehicle
MC	moisture content
SVR	support vector regression
